# Intrarenal Doppler ultrasonography reflects hemodynamics and predicts prognosis in patients with heart failure

**DOI:** 10.1038/s41598-020-79351-6

**Published:** 2020-12-17

**Authors:** Akiomi Yoshihisa, Koichiro Watanabe, Yu Sato, Shinji Ishibashi, Mitsuko Matsuda, Yukio Yamadera, Yasuhiro Ichijo, Tetsuro Yokokawa, Tomofumi Misaka, Masayoshi Oikawa, Atsushi Kobayashi, Yasuchika Takeishi

**Affiliations:** 1 Department of Cardiovascular Medicine, Fukushima Medical University, 1 Hikarigaoka, Fukushima, 960-1295 Japan; 2 Department of Clinical Laboratory Medicine, Fukushima Medical University, Fukushima, Japan

**Keywords:** Biomarkers, Cardiology, Diseases, Health care, Medical research

## Abstract

We aimed to clarify clinical implications of intrarenal hemodynamics assessed by intrarenal Doppler ultrasonography (IRD) and their prognostic impacts in heart failure (HF). We performed a prospective observational study, and examined IRD and measured interlobar renal artery velocity time integral (VTI) and intrarenal venous flow (IRVF) patterns (monophasic or non-monophasic pattern) to assess intrarenal hypoperfusion and congestion in HF patients (n = 341). Seven patients were excluded in VTI analysis due to unclear imaging. The patients were divided into groups based on (A) VTI: high VTI (VTI ≥ 14.0 cm, n = 231) or low VTI (VTI < 14.0 cm, n = 103); and (B) IRVF patterns: monophasic (n = 36) or non-monophasic (n = 305). We compared post-discharge cardiac event rate between the groups, and right-heart catheterization was performed in 166 patients. Cardiac index was lower in low VTI than in high VTI (P = 0.04), and right atrial pressure was higher in monophasic than in non-monophasic (P = 0.03). In the Kaplan–Meier analysis, cardiac event rate was higher in low VTI and monophasic groups (P < 0.01, respectively). In the Cox proportional hazard analysis, the combination of low VTI and a monophasic IRVF pattern was a predictor of cardiac events (P < 0.01). IRD imaging might be associated with cardiac output and right atrial pressure, and prognosis.

## Introduction

Heart failure (HF) is a widespread and serious problem that has been reported in many countries^1–3^, and causes multiple organ dysfunction through a combination of reduced arterial perfusion and passive congestion. The abdominal compartment, which contains the kidney, liver, splanchnic vasculature, gut, etc., has recently been focused upon in HF patients^4–9^. Central venous pressure (CVP) measured with right-heart catheterization (RHC) is a commonly used surrogate to evaluate organ congestion^10,11^. On the other hand, Nohria-Stevenson profiles demonstrated the clinical importance of assessments of perfusion (“cold” vs. “warm”), as well as congestion (“wet” vs. “dry”)^12^. However, these profiles are relatively subjective.

Renal and cardiac functions have close and complementary interconnections; this is called the cardio-renal syndrome (CRS)^13^. CRS is a disorder of the heart and kidneys whereby acute or chronic dysfunction in one organ may influence each other, and share common pathophysiology such as hemodynamics, neurohumoral, inflammatory, and oxidative injury^14^. Intrarenal congestion and hypoperfusion are critically involved in the pathophysiology of HF, and evaluation of renal function in HF patients is important^15,16^. Regarding renal congestion, it has recently been reported that intrarenal venous flow (IRVF) patterns (i.e. continuous, biphasic, or monophasic patterns) measured by intrarenal Doppler ultrasonography (IRD) are correlated with right atrial pressure (RAP), and a monophasic IRVF pattern was associated with higher RAP as well as worse outcome^17^. However, the usefulness of IRD to assess the clinical profiles, such as both hypoperfusion and congestion, has not been fully examined.

In the current study, we aimed to: (1) examine the intrarenal hemodynamics assessed by IRD (not only intrarenal congestion [IRVF patterns] but also intrarenal hypoperfusion [velocity time integral, VTI of interlobar renal artery]) in HF patients; and (2) clarify the clinical impacts of the IRD findings on prognosis in HF patients.

## Results

Comparisons of the high and low VTI groups are summarized in Table 1. The low VTI group was older, had a lower systolic blood pressure and higher heart rate, and had a higher prevalence of atrial fibrillation (AF), chronic kidney disease (CKD), higher levels of B-type natriuretic peptide (BNP), creatinine, urinary albumin creatinine ratio, and *N*-acetyl-β-d-glucosaminidase (NAG). In contrast, hemoglobin and estimated glomerular filtration rate (eGFR) were lower. The low VTI group also had higher levels of left atrial volume index (LAVI), early trans-mitral flow velocity to mitral annular velocity ratio (mitral valve E/e′), right atrial (RA) area, tricuspid regurgitation pressure gradient (TRPG), pulmonary artery pressure, RA pressure (RAP) and pulmonary artery wedge pressure (PAWP) and lower levels of left ventricular ejection fraction (LVEF), left ventricular outflow tract-VTI (LVOT-VTI), right ventricular fractional area change (RV-FAC) and cardiac index. In addition, there were significant associations between interlobar renal VTI and age, heart rate, presence of New York Heart Association (NYHA) class III or IV, AF, hypertension, CKD, anemia, levels of hemoglobin, BNP, creatinine, eGFR, urinary albumin creatinine ratio, urinary NAG, LVEF, LAVI, LVOT-VTI, mitral valve E/e′, RA area, RV area, RV-FAC, TRPG, pulmonary artery pressure, RAP and cardiac index. These results suggest that decreased VTI indicates intrarenal hypoperfusion, underlying low cardiac output, and leads to urinary albumin transudation or tubular damage.

**Table 1:** Comparisons of patient characteristics between interlobular renal artery VTI groups.

	*VTI group*			*Correlation with VTI*	
	High VTI (*n* = 231)	Low VTI (*n* = 103)	*P value*	*Correlation coefficient*	*P-value*
**Demographics**					
Age (years)	65.2 ± 13.0	69.6 ± 12.3	< 0.01	− 0.13	0.02
Male sex (n, %)	139 (60.2)	68 (66.0)	0.33	− 0.06	0.28
Body mass index (kg/m^2^)	24.0 ± 4.3	23.3 ± 5.3	0.25	0.07	0.18
Systolic blood pressure (mmHg)	120.1 ± 17.1	115.6 ± 17.8	0.03	0.11	0.05
Diastolic blood pressure (mmHg)	69.5 ± 12.4	68.4 ± 12.9	0.47	− 0.04	0.43
Heart rate (bpm)	68.5 ± 12.9	75.5 ± 15.0	< 0.01	− 0.38	< 0.01
NYHA class III or IV (n, %)	11 (4.8)	11 (10.7)	0.06	− 0.12	0.03
**Comorbidities**					
Atrial fibrillation (n, %)	71 (30.7)	49 (47.6)	< 0.01	− 0.07	< 0.01
Hypertension (n, %)	134 (58.0)	71 (68.6)	0.07	− 0.05	< 0.01
Dyslipidemia (n, %)	163 (70.6)	75 (72.8)	0.70	0.04	0.42
Diabetes mellitus (n, %)	74 (32.0)	37 (35.9)	0.53	− 0.02	0.74
Chronic kidney disease (n, %)	99 (42.9)	74 (71.8)	< 0.01	− 0.30	< 0.01
Anemia (n, %)	88 (38.1)	57 (55.3)	< 0.01	− 0.08	< 0.01
**Laboratory data**					
Hemoglobin (g/dL)	13.5 ± 1.9	12.9 ± 2.2	0.02	0.13	0.02
B-type natriuretic peptide (pg/ml)	57.7 (19.2–166.7)	257.6 (98.9–526.4)	< 0.01	− 0.28	< 0.01
Creatinine (mg/dL)	0.92 ± 0.37	1.26 ± 0.94	< 0.01	− 0.22	< 0.01
eGFR (mL/min/1.73 cm^2^)	62.4 ± 17.2	49.1 ± 16.5	< 0.01	0.31	< 0.01
Sodium (mEq/L)	139.7 ± 2.7	139.2 ± 3.5	0.14	0.10	0.06
Urinary protein creatinine ratio (g/g *Creatinine)	0 (0–0.06)	0 (0–0.25)	0.07	− 0.14	0.01
Urinary albumin creatinine ratio (mg/g *Creatinine)	11.5 (5.0–35.8)	25.0 (9–125.5)	0.04	− 0.21	< 0.01
Urinary β2 micro globulin (µg/mL)	0.11 (0.06–0.20)	0.12 (0.05–0.40)	0.99	− 0.08	0.18
Urinary NAG (U/L)	4.5 (2.4–8.7)	6.6 (3.1–14.6)	0.02	− 0.16	< 0.01
**Echocardiography**					
LVEF (%)	56.1 ± 15.4	49.3 ± 16.5	< 0.01	0.23	< 0.01
Left atrial volume index (mL/m^2^)	48.0 ± 27.7	64.4 ± 35.2	< 0.01	− 0.20	< 0.01
LVOT-VTI (cm)	18.2 ± 4.8	15.6 ± 5.3	< 0.01	0.28	< 0.01
Mitral valve E/e′	12.4 ± 8.0	15.7 ± 8.8	< 0.01	− 0.22	< 0.01
RA end systolic area (cm^2^)	17.2 ± 6.3	21.0 ± 7.2	< 0.01	− 0.25	< 0.01
RV area diastole (cm^2^)	19.6 ± 7.0	23.2 ± 9.3	0.04	− 0.26	< 0.01
RV area systole (cm^2^)	12.0 ± 5.5	16.4 ± 8.7	< 0.01	− 0.30	< 0.01
RV fractional area change (%)	40.0 ± 10.9	32.8 ± 12.5	< 0.01	0.32	< 0.01
Inferior vena cava diameter (mm)	15.1 ± 4.0	16.2 ± 4.8	0.07	− 0.07	0.22
TRPG (mmHg)	24.8 ± 12.7	29.5 ± 16.5	0.02	− 0.23	< 0.01
**Right-heart catherization**	n = 110	n = 56			
PAP mean (mmHg)	23.8 ± 9.8	29.5 ± 12.3	< 0.01	− 0.26	< 0.01
PAP systolic (mmHg)	35.5 ± 14.9	42.6 ± 20.5	< 0.01	− 0.24	< 0.01
PAP diastolic (mmHg)	14.9 ± 7.2	19.9 ± 8.6	< 0.01	− 0.29	< 0.01
Right atrial pressure mean (mmHg)	6.7 ± 3.7	8.5 ± 3.6	< 0.01	− 0.17	0.03
PAWP mean (mmHg)	13.5 ± 6.4	17.7 ± 8.8	< 0.01	− 0.18	0.02
Cardiac output (L/min)	4.22 ± 1.19	3.98 ± 1.02	0.16	0.13	0.09
Cardiac index (L/min/m^2^)	2.65 ± 0.64	2.36 ± 0.69	0.04	0.27	< 0.01
**Medication**					
RAS inhibitors (n, %)	180 (77.9)	77 (74.8)	0.57	− 0.18	0.53
β-Blockers (n, %)	128 (55.4)	76 (73.8)	< 0.01	0.31	< 0.01
Diuretics (n, %)	94 (40.7)	77 (74.8)	< 0.01	0.32	< 0.01
Inotropic agents (n, %)	14 (6.1)	16 (15.5)	< 0.01	0.28	< 0.01

*VTI* velocity time integral, *NYHA* New York Heart Association, *eGFR* estimated glomerular filtration rate, *NAG* *N*-acetyl-β-d-glucosaminidase, *LVEF* left ventricular (LV) ejection fraction, *LVOT* LV outflow tract, *RA* right atrial, *RV* right ventriclar, *TRPG* tricuspid regurgitation pressure gradient, *PAP* pulmonary artery pressure, *PAWP* pulmonary artery wedge pressure, *RAS* renin-angiotensin system.

Comparisons of the non-monophasic and monophasic groups are summarized in Table 2. The monophasic group were older, had a higher prevalence of AF, higher levels of BNP and lower levels of eGFR, higher levels of LAVI, mitral valve E/e′, RA area, inferior vena cava diameter and TR-PG, and higher levels of RAP. In contrast, blood pressure, prevalence of hypertension, dyslipidemia, levels of creatinine, sodium, or cardiac index did not significantly differ between the groups. In addition, there were significant associations between monophasic IRVF pattern and age, presence of AF, levels of BNP, eGFR, LAVI, mitral valve E/e′, RA area, inferior vena cava diameter, TRPG and RAP. These results suggest that a monophasic IRVF pattern indicates intrarenal congestion, and underlying increased CVP, RAP or right heart volume overload.

**Table 2:** Comparisons of patient characteristics among IRVF patterns.

	*IRVF pattern group*			*Correlation with monophasic pattern*	
	Non-monophasic (*n* = 305)	Monophasic (*n* = 36)	*P value*	*Correlation coefficient*	*P value*
**Demographics**					
Age (years)	65.9 ± 12.9	72.0 ± 11.8	< 0.01	0.05	< 0.01
Male sex (n, %)	196 (64.3)	17 (47.2)	0.07	− 0.70	0.06
Body mass index (kg/m^2^)	23.9 ± 4.7	22.4 ± 3.6	0.05	− 0.09	0.05
Systolic blood pressure (mmHg)	119.0 ± 17.2	117.1 ± 19.3	0.55	− 0.01	0.54
Diastolic blood pressure (mmHg)	69.6 ± 12.3	66.7 ± 14.2	0.20	− 0.02	0.19
Heart rate (bpm)	71.0 ± 13.8	67.8 ± 14.7	0.20	− 0.02	0.19
NYHA class III or IV (n, %)	21 (6.9)	2 (5.6)	1.00	− 0.23	0.76
**Comorbidities**					
Atrial fibrillation (n, %)	104 (34.1)	21 (58.3)	< 0.01	0.99	< 0.01
Hypertension (n, %)	183 (60.0)	24 (66.7)	0.48	0.29	0.44
Dyslipidemia (n, %)	217 (71.1)	23 (63.9)	0.44	− 0.33	0.37
Diabetes mellitus (n, %)	101 (33.1)	11 (30.6)	0.85	− 0.19	0.76
Chronic kidney disease (n, %)	154 (50.5)	24 (66.7)	0.08	0.67	0.07
Anemia (n, %)	130 (42.6)	20 (55.6)	0.16	0.52	0.14
**Laboratory data**					
Hemoglobin (g/dL)	13.4 ± 2.0	12.7 ± 2.1	0.06	− 0.17	0.06
B-type natriuretic peptide (pg/ml)	86.4 (23.5–251.8)	218.9 (142.9–547.9)	< 0.01	0.01	0.01
Creatinine (mg/dL)	1.06 ± 0.78	1.04 ± 0.39	0.87	− 0.04	0.87
eGFR (mL/min/1.73 cm^2^)	58.8 ± 19.3	52.1 ± 15.0	0.04	− 0.02	0.04
Sodium (mEq/L)	139.5 ± 2.9	139.9 ± 3.6	0.50	0.04	0.50
Urinary protein creatinine ratio (g/g *Creatinine)	0 (0–0.09)	0 (0–0.203)	0.54	− 0.17	0.69
Urinary albumin creatinine ratio (mg/g *Creatinine)	14.5 (6–42.8)	18.5 (8–87.5)	0.67	0.01	0.75
Urinary β2 micro globulin (µg/mL)	011 (0.05–0.24)	0.12 (0.05–0.28)	0.49	− 0.02	0.69
Urinary NAG (U/L)	4.8 (2.4–9.7)	6.2 (3.8–15.0)	0.35	0.01	0.29
**Echocardiography**					
LVEF (%)	54.5 ± 15.3	49.2 ± 19.5	0.06	− 0.02	0.06
Left atrial volume index (mL/m^2^)	51.0 ± 29.4	74.7 ± 38.0	< 0.01	0.02	< 0.01
LVOT-VTI (cm)	17.3 ± 5.0	17.3 ± 6.0	0.99	0.01	0.99
Mitral valve E/e′	12.8 ± 7.8	18.8 ± 10.9	< 0.01	0.06	< 0.01
RA end-systolic area (cm^2^)	17.8 ± 6.7	23.1 ± 7.3	< 0.01	0.10	< 0.01
RV area diastole (cm^2^)	20.6 ± 7.7	20.9 ± 8.8	0.88	0.01	0.87
RV area systole (cm^2^)	13.1 ± 6.6	14.0 ± 8.4	0.63	0.02	0.63
RV fractional area change (%)	38.0 ± 11.5	35.1 ± 13.9	0.34	− 0.02	0.34
Inferior vena cava diameter (mm)	15.3 ± 4.3	17.0 ± 4.2	0.03	0.09	0.03
TRPG (mmHg)	25.4 ± 12.6	34.3 ± 21.6	< 0.01	0.03	< 0.01
**Right-heart catherization**	n = 139	n = 27			
PAP mean (mmHg)	25.3 ± 10.9	29.2 ± 11.6	0.11	0.03	0.10
PAP systolic (mmHg)	37.4 ± 16.6	41.8 ± 20.8	0.23	0.01	0.24
PAP diastolic (mmHg)	16.3 ± 8.0	19.0 ± 8.0	0.31	0.04	0.12
Right atrial pressure mean (mmHg)	7.2 ± 3.9	9.0 ± 2.7	0.03	0.10	0.02
PAWP mean (mmHg)	14.7 ± 7.7	16.6 ± 6.0	0.21	0.03	0.21
Cardiac output (L/min)	4.19 ± 1.20	4.07 ± 1.15	0.65	− 0.08	0.65
Cardiac index (L/min/m^2^)	2.59 ± 0.68	2.66 ± 0.59	0.60	0.16	0.59
**Medication**					
RAS inhibitors (n, %)	234 (76.7)	30 (83.3)	0.53	0.42	0.37
β-Blockers (n, %)	183 (60.0)	25 (69.4)	0.37	0.42	0.27
Diuretics (n, %)	147 (48.2)	28 (77.8)	< 0.01	0.87	< 0.01
Inotropic agents (n, %)	29 (9.5)	1 (2.8)	0.34	0.27	0.21

*IRVF* intrarenal venous flow, *NYHA* New York Heart Association, *Egfr* estimated glomerular filtration rate, *NAG* *N*-acetyl-β-d-glucosaminidase, *LVEF* left ventricular (LV) ejection fraction, *LVOT* LV outflow tract, *VTI* velocity time integral, *RA* right atrial, *RV* right ventricular, *TRPG* tricuspid regurgitation pressure gradient, *PAP* pulmonary artery pressure, *PAWP* pulmonary artery wedge pressure, *RAS* renin-angiotensin system.

**Figure 1:**
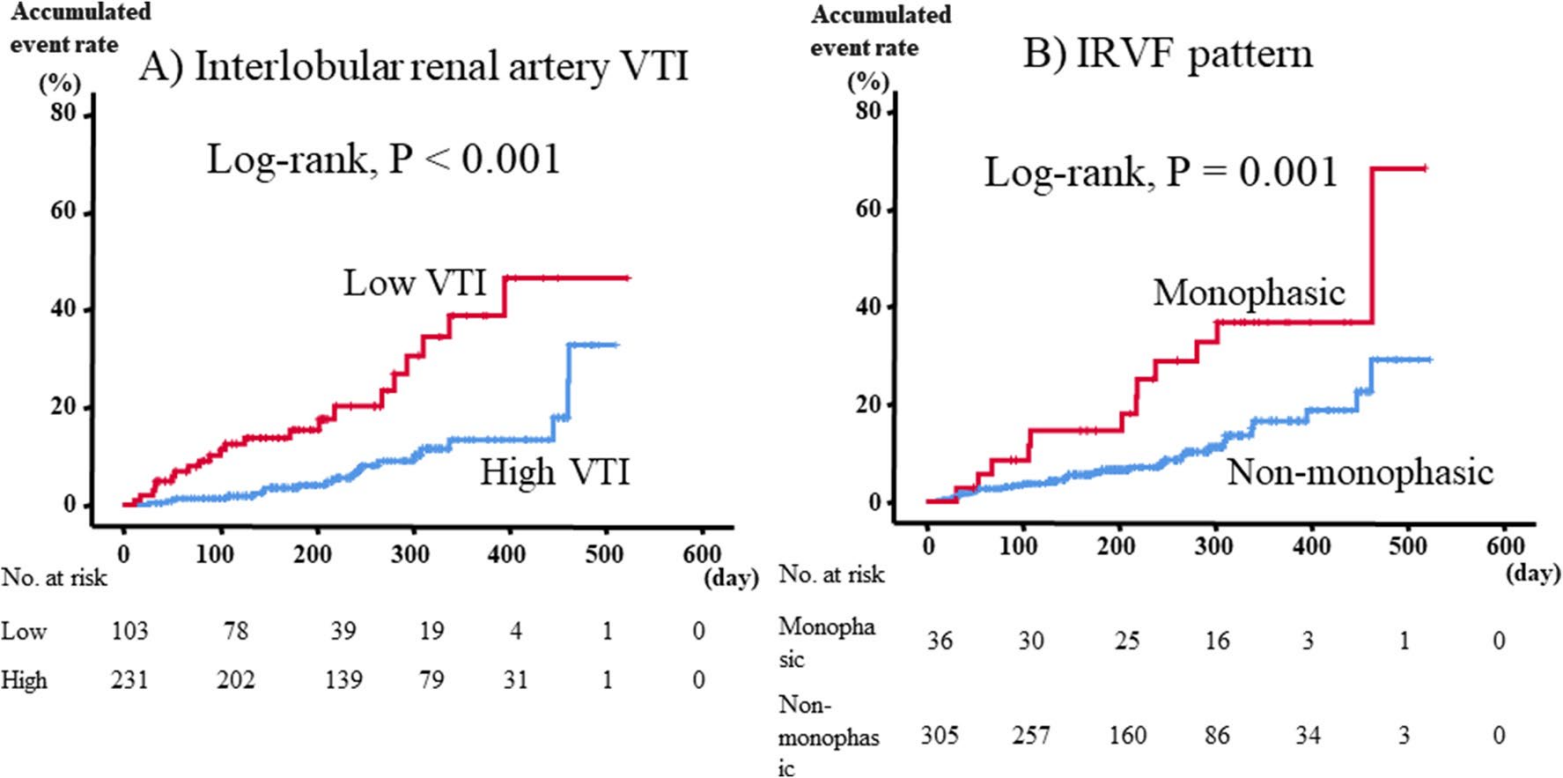
Kaplan–Meier analysis for cardiac events. *VTI* velocity time integral, *IRVF* intrarenal venous flow.

**Figure 2:**
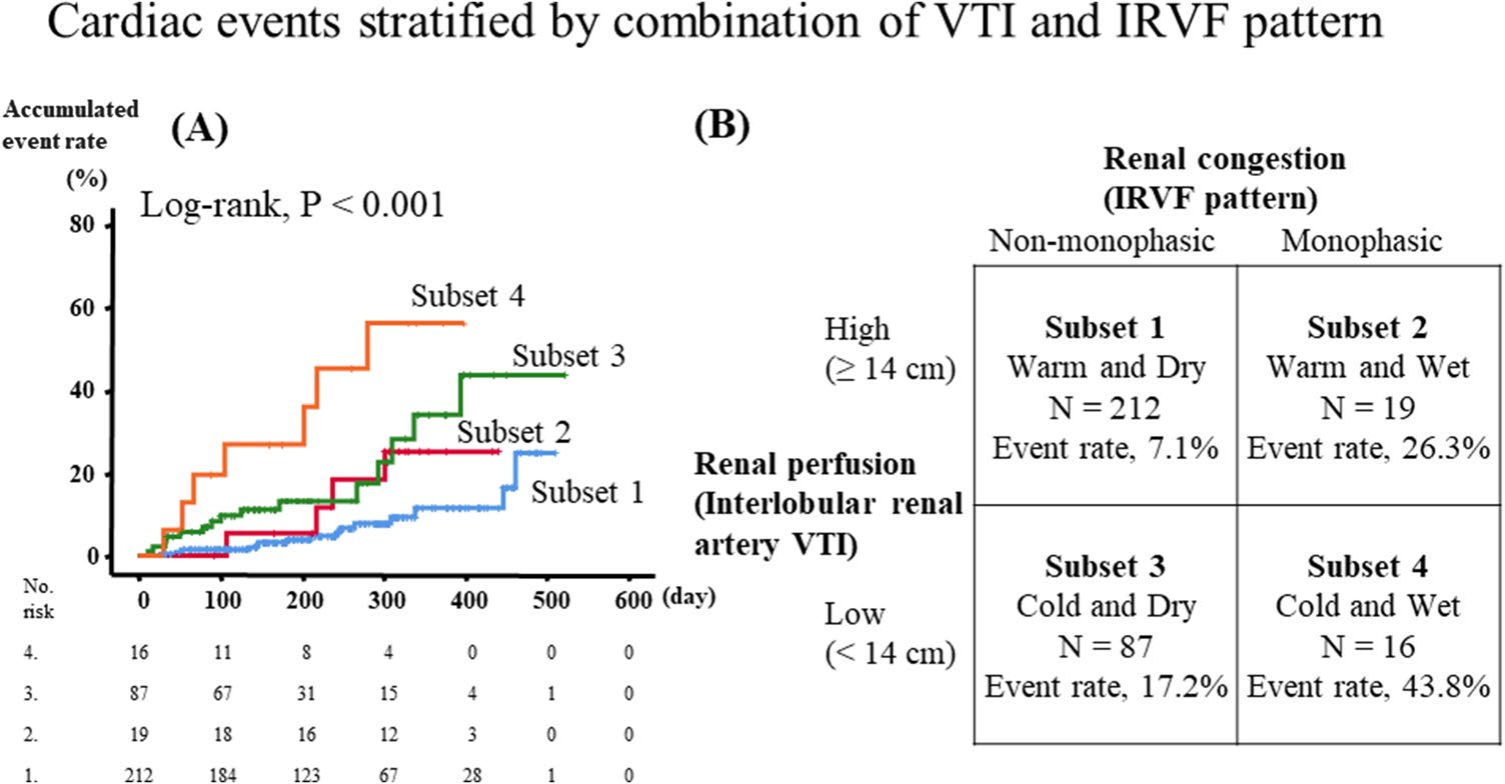
Cardiac events stratified by combination of VTI and IRVF pattern. *VTI* velocity time integral, *IRVF* intrarenal venous flow.

During the follow up period (mean 231 ± 122 days, range 12–523 days), 42 cardiac events including eight cardiac deaths and 34 worsening HF occurred. As shown in Fig. 1, in the Kaplan–Meier analysis, cardiac event rates were significantly higher in the low VTI group than in the high VTI group (log-rank P < 0.01), as well as in the monophasic group than in the non-monophasic group (log-rank P < 0.01). In addition, as shown in Fig. 2, HF patients with low VTI and a monophasic IRVF pattern (subset 4) had the highest cardiac event rate (log-rank P < 0.01). In the univariate Cox proportional hazard analysis (Table 3), low VTI and a monophasic IRVF pattern were associated with a high cardiac event rate (low VTI, hazard ratio [HR] 3.65, 95% CI 1.97–6.75; monophasic IRVF pattern, HR 2.86, 95% CI 1.46–5.59), and the combination of low VTI and a monophasic IRVF pattern (vs. high VTI and non-monophasic patterns as reference) was a predictor of cardiac events in HF patients (HR 8.36, 95% CI 3.37–20.75). These results suggest that the combination of interlobar renal VTI (intrarenal perfusion) and IRVF patterns (intrarenal congestion) might be a useful tool for evaluating renal hemodynamic subsets and estimating prognosis. With regard to the multivariate Cox proportional hazard analysis, as there were limited number of events (42 events) and multicollinearity between IRD findings and possible confounding factors such as parameters of demographics, comorbidities, laboratory data, echocardiography and right-heart catherization, we only adjusted for age and sex. Even after adjusting for age and sex, low VTI, a monophasic IRVF pattern and combinations of low VTI and a monophasic IRVF pattern still remain associated with high cardiac event rates.

**Table 3:** Cox proportional hazard model of cardiac events (factors in parameters of renal ultrasonography).

Cardiac event (42 events/334 patients)	HR	95% CI	P value
**VTI group**			
VTI (low vs. high)	3.65	1.97–6.75	< 0.01
VTI (low vs. high)*	3.06	1.62–5.78	< 0.01
**IRVF pattern group**			
IRVF pattern (monophasic vs. non-monophasic)	2.86	1.46–5.59	< 0.01
IRVF pattern (monophasic vs. non-monophasic)*	2.25	1.11–4.55	0.02
**Combination of VTI and IRVF pattern**			
Subset 1 (high VTI and non-monophasic IRVF)	Reference	–	–
Subset 2 (high VTI and monophasic IRVF)	2.92	1.06–8.07	0.04
Subset 2 (high VTI and monophasic IRVF)*	2.09	0.74–5.93	0.16
Subset 3 (low VTI and non-monophasic IRVF)	3.57	1.73–7.38	< 0.01
Subset 3 (low VTI and non-monophasic IRVF)*	2.92	1.39–6.14	< 0.01
Subset 4 (low VTI and monophasic IRVF)	8.36	3.37–20.75	< 0.01
Subset 4 (low VTI and monophasic IRVF)*	6.47	2.50–16.77	< 0.01

*HR* hazard ratio, *CI* confidence interval, *VTI* velocity time integral, *IRVF* intrarenal venous flow.

*Adjusted: adjusted for age and sex.

Intrarenal congestion and hypoperfusion determined by IRD (IRVF patterns and interlobar renal artery VTI) are associated with adverse prognosis in HF patients.

## Discussion

To the best of our knowledge, the present study was the first to report that (1) the associations between parameters of not only intrarenal congestion (IRVF patterns), but also of intrarenal hypoperfusion (interlobular renal artery VTI) determined by IRD and both RHC and echocardiography, and that (2) prognostic impacts of intrarenal congestion and hypoperfusion in patients with HF. The combination of interlobar renal VTI and IRVF patterns might be a useful tool for evaluating intrarenal hemodynamic subsets such as the Nohria–Stevenson profiles, and may be therapeutic targets.

Intrarenal congestion caused by increased CVP, namely increased right-sided pressure overload, has been one of the main pathophysiologic findings in CRS^10,11,18–21^. High CVP, rather than low cardiac output, predetermines the risk of worsening renal function in decompensated HF^10^. Previous studies have shown that IRVF patterns were associated with increased RAP levels, and correlated with clinical outcomes^17,22^. The increased CVP causes the increase of renal interstitial pressure and intrarenal parenchymal compliance around the intrarenal vessels, fibrosis, and leads the resistance of IRVF^17,23,24^. It has been reported that 22.5% of HF patients showed monophasic IRVF pattern^17^, and that the IRVF patterns could be changed depending on the renal congestion, and their changes were associated with renal impairment and poor prognosis^25^. In the present study, prevalence of monophasic IRVF pattern was 10.6%, and was lower than 22.5% of previous study^17^. Although we could not fully explain the difference of their IRVF patterns, above changes in IRVF patterns^25^ depending on renal congestion might affect their differences. Increased renal interstitial pressure reduces hydrostatic and colloid osmotic pressure differences between glomerular capillaries and Bowman’s space^26^. In addition, systemic venous congestion raises neurohormonal activation (e.g. renin-angiotensin system) resulting in renal vasoconstriction^27,28^. Neurohormonal activation reduces the GFR^29,30^, decreases plasma natriuretic peptide^31^, leads to HF progression, may contribute to multiple organ failure^11,32^, and results in adverse prognosis^33^.

Regarding intrarenal hypoperfusion, the results of the current study suggest that low interlobar renal artery VTI was associated with low cardiac index, low systolic blood pressure, and low eGFR. Hypotension leads to intrarenal hypoperfusion^34^. In pre-renal acute kidney injury, when renal hypoperfusion is sustained, eGFR is initially decreased without parenchymal damage^35^. The sustained inadequate oxygen and nutrient delivery to the nephrons and the adenosine triphosphate depletion activates epithelial cellular injury and death via necrosis or apoptosis, or both, which ultimately leads to endothelial injury, activation of inflammatory processes, and renal dysfunction^35^. Subsequently, afferent glomerular arterial vasoconstriction occurs and secretes renin, which further activates the renin–angiotensin system and results in efferent glomerular arterial vasoconstriction^15^.

The current study reported the associations between impaired intrarenal perfusion and increased intrarenal congestion, as well as both cardiac function and prognosis. The combination of interlobar renal VTI and IRVF patterns might be a useful tool for evaluating renal hemodynamic subsets such as the Nohria–Stevenson profiles, and may be a therapeutic indicator for managing organ perfusion and congestion.

Study limitations are as follows: first, the number of patients was relatively small and the follow-up period was comparatively short, because the study was carried out in a single center. We could not fully adjust confounding factors in the Cox proportional hazard analysis. Second, although HF patients with renal artery stenosis, dialysis or renal atrophy were excluded, we were unable to completely exclude the presence of subclinical renal diseases. Third, since we conducted the present study using variables measured only during hospitalization, changes in variables (e.g. interlobar renal VTI, IRVF patterns) after discharge were not examined. Fourth, since attending physician decided performing RHC, there might be potential selection bias. Therefore, the present results should be viewed as preliminary, and further studies with a larger population and longer follow up period are needed.

## Methods

### Subjects and study protocol

This was a prospective observational study of a total of 380 decompensated HF patients, who had undergone abdominal ultrasonography and were discharged from Fukushima Medical University Hospital between April 2018 and March 2019. The diagnosis of HF was defined by cardiologists based on the Framingham criteria, characterized by typical symptoms (e.g. breathlessness and fatigue) and accompanied signs (e.g. elevated jugular venous pressure, pulmonary crackles and peripheral edema). Cardiologists decided needs of hospitalization due to decompensated and/or worsening heart failure in all cases, when managements were necessary such as intravenous agents, respiratory care, dialysis, mechanical support, and etc^1–3^. Blood samples, abdominal ultrasonography and echocardiography were obtained at hospital discharge. Patients with poor quality of images from abdominal ultrasonography (n = 4), renal artery stenosis (n = 28), and/or end-stage renal disease receiving dialysis or renal atrophy (n = 7) were excluded. Of these 341 patients, RHC was partly performed in 166, among whom, in order to assess the interlobar renal artery VTI that could predict preserved cardiac index (≥ 2.2 L/min/m^2^), we measured the area under the curve of the receiver operating curve. A cut-off value with interlobar renal artery VTI of ≥ 14.0 cm predicted cardiac index (≥ 2.2 L/min/m^2^) with an area under the curve of 0.616 (95% confidence interval [CI] 0.508–0.696), with a sensitivity of 0.704 and specificity of 0.600. Of these 341 patients, 7 patients were excluded in VTI analysis due to unclear imaging. Finally, these patients were divided into groups based on A) interlobar renal artery VTI: high VTI group (VTI ≥ 14.0 cm, n = 231) or low VTI group (VTI < 14.0 cm, n = 103) and B) IRVF pattern: monophasic group (n = 36) or non-monophasic group (n = 305).

First, we compared the clinical features and results from laboratory tests, echocardiography and RHC between the groups. In addition, we performed a correlation analysis of associations between levels of both of interlobar renal artery VTI or IRVF patterns, and parameters of laboratory tests, echocardiography, and RHC. Second, the patients were followed up until December 2019 for cardiac events as composites of cardiac death or unplanned re-hospitalization due to decompensated HF. For patients that experienced two or more events, only the first event was included in the analysis. Since these patients visited patient’s referring hospital monthly or bi-monthly, status and dates of death were obtained from the patient’s medical records. If these data were unavailable, status was ascertained by a telephone call to the patient’s referring hospital physician. This study complied with the Declaration of Helsinki and the STROBE (Strengthening the Reporting of Observational Studies in Epidemiology) statement^36,37^. The study protocol was approved by the Ethics Committee of Fukushima Medical University. All patients gave written informed consent. Informed consent from next of kin or legally authorized representatives is provided.

### IRD analysis, interlobuar renal artery VTI and IRVF pattern

**Figure 3:**
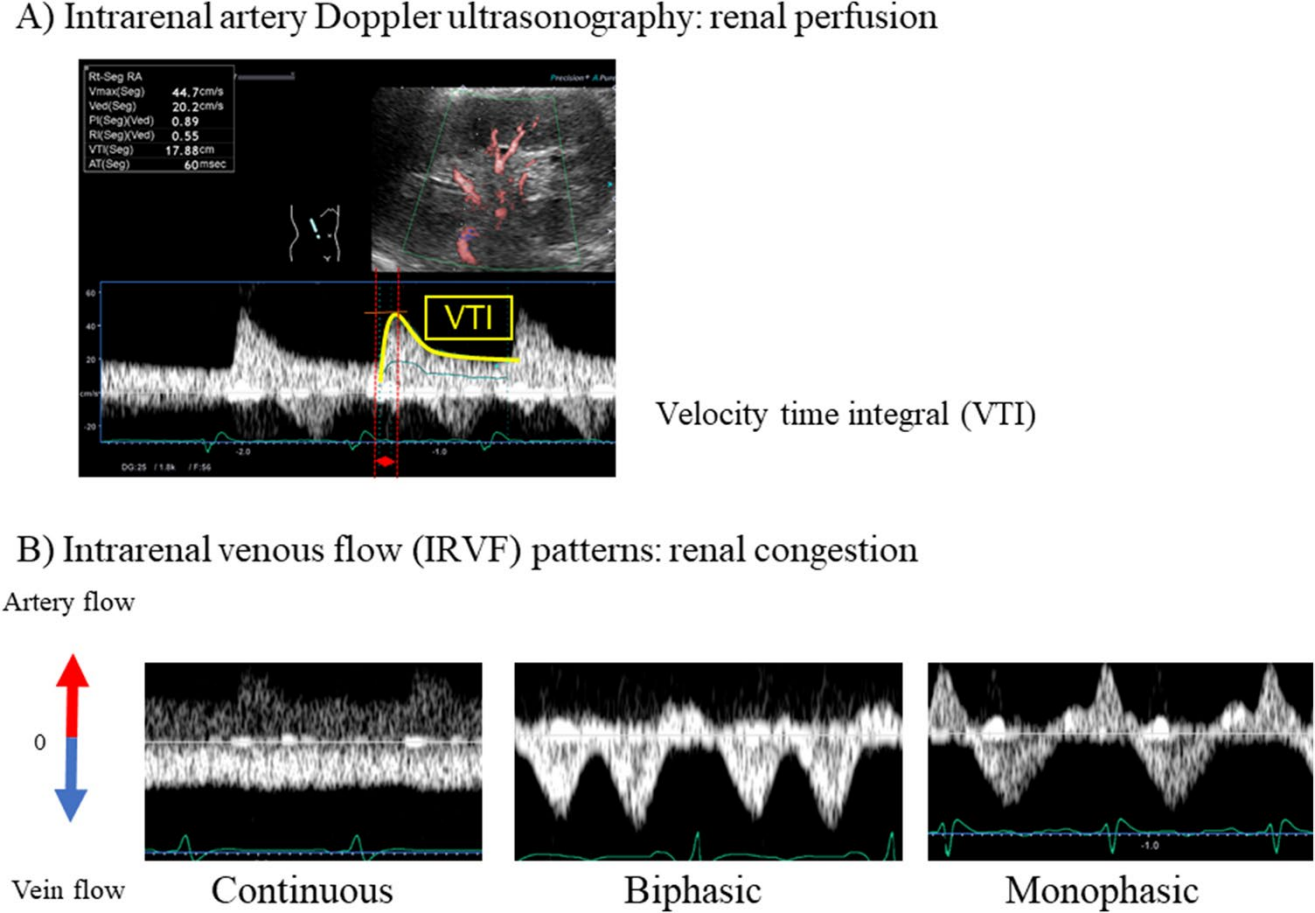
Abdominal ultrasonography.

Abdominal ultrasonography was performed by two experienced sonographers, who were blinded to all clinical data before discharge using an ultrasound system (Aplio i800, Canon Medical Systems, Tochigi, Japan) equipped with a wideband convex i8CX1 multifrequency probe (central frequency 4.0 MHz, range 1.8–6.4 MHz). Patients fasted for at least 12 h before the examination. IRD was recorded in the right kidney with the patient in the left lateral decubitus position. Color Doppler images were used to determine interlobar vessels. The velocity range of the color Doppler was set to approximately 12–16 cm/s in order to determine interlobar vessels, and the sample volume was set based on the color Doppler signals derived from interlobar vessels^17^. Pulsed Doppler waveforms of the interlobar arteries and veins were recorded simultaneously. As shown in Fig. 3, we examined (a) VTI as a marker of intrarenal hypoperfusion, and (b) IRVF patterns as a marker of intrarenal congestion. The spectral Doppler renal blood flow velocities were recorded, and the VTI was measured as the area under the outermost portion of the spectral velocity envelope^38^. Doppler waveforms of IRVF were divided into three flow patterns: continuous, biphasic discontinuous, and monophasic discontinuous (Fig. 3)^17,39^. IRVF patterns are reported to be altered by increases in RAP and are affected by both CVP and reduced renal parenchymal compliance related to intrarenal congestion^17^. It has been reported that monophasic IRVF pattern is especially associated with increased RAP^17^; thus, we focused on monophasic or non-monophasic (continuous-biphasic) IRVF patterns in the present study.

### Echocardiography

Patients underwent echocardiography performed by experienced echocardiographers using standard techniques as previously reported^40^. The echocardiographic parameters included LVEF, left atrial volume, LVOT-VTI, mitral valve E/e′, right atrium area, RV-FAC, inferior vena cava diameter, and TRPG. All measurements were performed using ultrasound systems (ACUSON Sequoia, Siemens Medical Solutions USA, Inc., Mountain View, CA, USA)^40^.

### RHC and hemodynamic measurements

Of the 341 patients, RHC was partly performed based on remedial judgment of the attending physician in 166 patients. RHC was performed with in three days of abdominal ultrasonography, with the patients in a stable condition without changes in medications including doses, similar to setting of abdominal ultrasonography. All RHC was performed as previously reported^41^.

### Statistical analysis

Normally distributed data are expressed as mean ± standard deviation. Non-normally distributed data are presented as median (interquartile range). The categorical variables are expressed as numbers (percentages), and the chi-square test was performed for its comparison. For the comparisons of parametric and non-parametric variables, Student’s t test and the Mann–Whitney U test were used, respectively. Associations between VTI and the parameters of laboratory data, echocardiography or RHC, were examined using Pearson’s correlation analysis for parametric variables, Spearman’s correlation analysis for non-parametric variables. Logistic regression analysis to determine the categorical variables associated with the interlobar renal artery VTI. Logistic regression analysis was also performed to determine the associations between monophasic IRVF pattern and other variables. Kaplan–Meier analysis was used with a log-rank test to assess cardiac event rates. These curves helped in identifying non-proportionality patterns in hazard function such as convergence (difference in risk between the groups decreases with time), divergence, or crossing of the curves. In addition, proportional hazard assumptions were confirmed by log–log analysis. We assessed IRVF patterns and interlobar renal artery VTI levels as predictors for post-discharge cardiac events using the univariate Cox proportional hazard analysis with only age and sex adjusted. Because of small number of events and sample size, as well as the presence of multicollinearity, we did not perform multivariate Cox proportional hazard analyses. In addition, we further classified patients into four subsets in accordance with Nohria–Stevenson clinical profiles: subset 1, high VTI and non-monophasic IRVF patterns (warm-dry); subset 2, high VTI and a monophasic IRVF pattern (warm–wet); subset 3, low VTI and non-monophasic patterns (cold–dry); and subset 4, low VTI and a monophasic IRVF pattern (cold–wet). The predictive value of classification was assessed using the Kaplan–Meier analysis and the Cox proportional hazard analysis. A value of P < 0.05 was considered statistically significant for all comparisons. These analyses were performed using SPSS ver. 26.0 (IBM, Armonk, NY, USA).

## Data Availability

The data that support the findings of this study are available from the corresponding author upon reasonable request.
